# Aqua­bis(nicotinamide-κ*N*)(thio­cyanato-κ*N*)copper(II)

**DOI:** 10.1107/S1600536807068511

**Published:** 2008-01-09

**Authors:** Chunyuan Li, Weijia Ding, Changlun Shao

**Affiliations:** a Department of Applied Chemistry, College of Science, South China Agricultural University, Guangzhou 510642, People’s Republic of China; b School of Medicine and Pharmacy, Ocean University of China, Qingdao 266003, People’s Republic of China

## Abstract

In the title compound, [Cu(NCS)_2_(C_6_H_6_N_2_O)_2_(H_2_O)], the Cu atom adopts a square-based pyramidal CuN_4_O coordination, with the water O atom in the apical position. The pairs of N-bonded nicotinamide ligands and thio­cyanate anions in the basal plane are in a *trans* configuration. In the crystal structure, the mol­ecules are connected into sheets by N—H⋯O and O—H⋯O hydrogen bonds.

## Related literature

For related literature, see: Beatty (2001 ▶); Aakeröy *et al.* (2004 ▶).
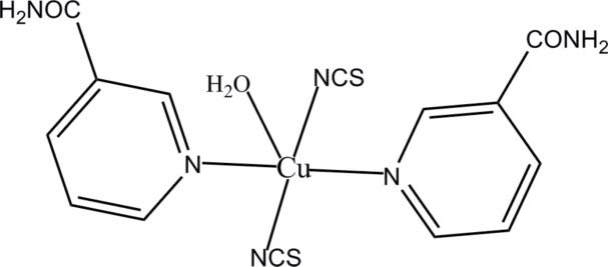


## Experimental

### 

#### Crystal data

[Cu(NCS)_2_(C_6_H_6_N_2_O)_2_(H_2_O)]
*M* *_r_* = 441.97
Monoclinic, 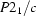

*a* = 11.078 (5) Å
*b* = 8.950 (4) Å
*c* = 18.702 (9) Å
β = 90.333 (8)°
*V* = 1854.3 (15) Å^3^
*Z* = 4
Mo *K*α radiation
μ = 1.43 mm^−1^
*T* = 293 (2) K
0.42 × 0.35 × 0.30 mm

#### Data collection

Bruker SMART CCD diffractometer
Absorption correction: multi-scan (*SADABS*; Bruker, 1997 ▶) *T*_min_ = 0.542, *T*_max_ = 0.663
10592 measured reflections
4041 independent reflections
3292 reflections with *I* > 2σ(*I*)
*R*_int_ = 0.019

#### Refinement

*R*[*F* ^2^ > 2σ(*F* ^2^)] = 0.031
*wR*(*F* ^2^) = 0.090
*S* = 1.07
4041 reflections
236 parameters
H-atom parameters constrained
Δρ_max_ = 0.35 e Å^−3^
Δρ_min_ = −0.36 e Å^−3^

### 

Data collection: *SMART* (Bruker, 1997 ▶); cell refinement: *SAINT* (Bruker, 1997 ▶); data reduction: *SAINT*; program(s) used to solve structure: *SHELXS97* (Sheldrick, 2008 ▶); program(s) used to refine structure: *SHELXL97* (Sheldrick, 2008 ▶); molecular graphics: *SHELXTL* (Bruker, 1997 ▶); software used to prepare material for publication: *SHELXTL*.

## Supplementary Material

Crystal structure: contains datablocks global, I. DOI: 10.1107/S1600536807068511/hb2684sup1.cif

Structure factors: contains datablocks I. DOI: 10.1107/S1600536807068511/hb2684Isup2.hkl

Additional supplementary materials:  crystallographic information; 3D view; checkCIF report

## Figures and Tables

**Table 1:** Selected bond lengths (Å)

Cu1—N6	1.955 (2)
Cu1—N5	1.969 (2)
Cu1—N1	2.049 (2)
Cu1—N3	2.058 (2)
Cu1—O3	2.442 (4)

**Table 2:** Hydrogen-bond geometry (Å, °)

*D*—H⋯*A*	*D*—H	H⋯*A*	*D*⋯*A*	*D*—H⋯*A*
O3—H3*A*⋯O2^i^	0.88	1.94	2.815 (3)	171
O3—H3*B*⋯O2^ii^	0.86	2.00	2.848 (3)	172
N2—H2*A*⋯O3^iii^	0.86	2.09	2.944 (3)	176
N4—H4*B*⋯O1^iv^	0.86	2.05	2.857 (3)	157

Symmetry codes: (i) 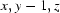; (ii) 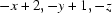; (iii) 
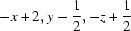
; (iv) 
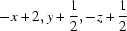
.

**Fig. 1.**
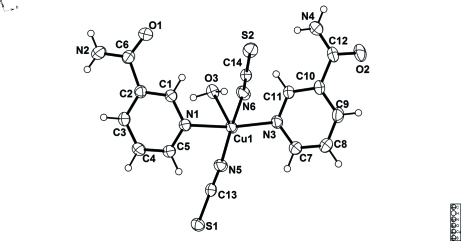
The molecular structure of (I) with displacement ellipsoids drawn at the 40% probability level (arbitrary spheres for the H atoms).

**Fig. 2.**
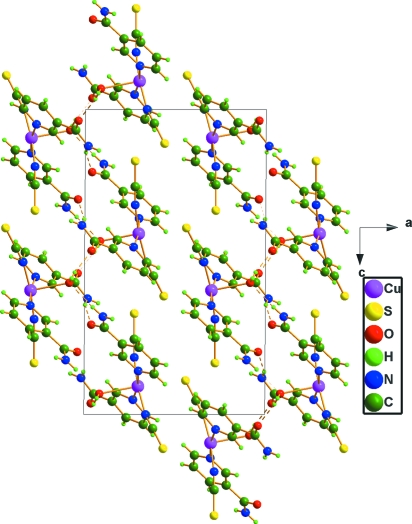
The layered hydrogen-bonded network in (I) viewed down the b axis direction. Hydrogen bonds are shown as dashed lines.

## supplementary crystallographic information

### Comment

Due to their inherent coordination and hydrogen bonding donor/acceptor functionalities, nicotinamide ligands have been used in crystal engineering to construct extended frameworks sustained both by hydrogen bonds and coordination bonds (Beatty 2001; Christer *et al.*, 2004). In this paper, we report the synthesis and crystal structure of the title compound, (I).

In compound (I), the metal center occupies a general position, and is coordinated with four nitrogen atoms from two *trans*-nicotinamide ligands and two *trans*-NCS anions in a square-planar geometry, as shown in Fig 1. The amide moieties are oriented in same directions. The two pyridine rings coordinated to the Cu centre are twisted by 3.63 (2)°. The distance between Cu center and the O atom of the aqua ligand is 2.442 (4) Å, which suggests a weak non-covalent interaction (Table 1). The Cu complex units are connected *via* N—H···O hydrogen bonds in a head-to-head fashion, resulting in chains in the crystal. The chains are further linked *via* O—H···O hydrogen bonds between the coordinated water molecules and amide groups to lead to infinite sheets, as shown in Fig 2.

### Experimental

CuCl_2_.6H_2_O (1 mmol), nicotinamide (2 mmol) and NaNCS (1 mmol) were dissolved in water and blue blocks of (I) were obtained by slow evaporation at room temperature about 5 days in 82% yield.

### Refinement

The H atoms attached to C or N atoms were placed in idealized positions (C—H = 0.93 Å, N–H = 0.86 Å), and refined as riding with *U*_iso_(H) = 1.2*U*_eq_(C or N).

The O-bound H atoms were located in difference maps and refined as riding in their as-found relative positions with *U*_iso_(H) = 1.2*U*_eq_(O).

### Crystal data

**Table:**

[Cu(NCS)_2_(C_6_H_6_N_2_O)_2_(H_2_O)]	*F*_000_ = 900
*M**_r_* = 441.97	*D*_x_ = 1.583 Mg m^−^^3^
Monoclinic, *P*2_1_/*c*	Mo *K*α radiation λ = 0.71073 Å
Hall symbol: -P 2ybc	Cell parameters from 10592 reflections
*a* = 11.078 (5) Å	θ = 12–18º
*b* = 8.950 (4) Å	µ = 1.43 mm^−^^1^
*c* = 18.702 (9) Å	*T* = 293 (2) K
β = 90.333 (8)º	Block, blue
*V* = 1854.3 (15) Å^3^	0.42 × 0.35 × 0.30 mm
*Z* = 4	

### Data collection

**Table:**

Bruker SMART CCD diffractometer	4041 independent reflections
Radiation source: fine-focus sealed tube	3292 reflections with *I* > 2σ(*I*)
Monochromator: graphite	*R*_int_ = 0.019
*T* = 293(2) K	θ_max_ = 27.2º
ω scans	θ_min_ = 2.5º
Absorption correction: Multi-Scan(SADABS; Bruker, 1997)	*h* = −14→14
*T*_min_ = 0.542, *T*_max_ = 0.663	*k* = −11→8
10592 measured reflections	*l* = −23→23

### Refinement

**Table:**

Refinement on *F*^2^	Hydrogen site location: difmap and geom
Least-squares matrix: full	H-atom parameters constrained
*R*[*F*^2^ > 2σ(*F*^2^)] = 0.031	*w* = 1/[σ^2^(*F*_o_^2^) + (0.045*P*)^2^ + 0.9888*P*] where *P* = (*F*_o_^2^ + 2*F*_c_^2^)/3
*wR*(*F*^2^) = 0.090	(Δ/σ)_max_ < 0.001
*S* = 1.07	Δρ_max_ = 0.35 e Å^−^^3^
4041 reflections	Δρ_min_ = −0.36 e Å^−^^3^
236 parameters	Extinction correction: SHELXL97 (Sheldrick, 2008), Fc^*^=kFc[1+0.001xFc^2^λ^3^/sin(2θ)]^-1/4^
Primary atom site location: structure-invariant direct methods	Extinction coefficient: 0.0017 (5)
Secondary atom site location: difference Fourier map	

### Special details

**Table:**

Geometry. All e.s.d.'s (except the e.s.d. in the dihedral angle between two l.s. planes) are estimated using the full covariance matrix. The cell e.s.d.'s are taken into account individually in the estimation of e.s.d.'s in distances, angles and torsion angles; correlations between e.s.d.'s in cell parameters are only used when they are defined by crystal symmetry. An approximate (isotropic) treatment of cell e.s.d.'s is used for estimating e.s.d.'s involving l.s. planes.
Refinement. Refinement of *F*^2^ against ALL reflections. The weighted *R*-factor *wR* and goodness of fit *S* are based on *F*^2^, conventional *R*-factors *R* are based on *F*, with *F* set to zero for negative *F*^2^. The threshold expression of *F*^2^ > σ(*F*^2^) is used only for calculating *R*-factors(gt) *etc*. and is not relevant to the choice of reflections for refinement. *R*-factors based on *F*^2^ are statistically about twice as large as those based on *F*, and *R*- factors based on ALL data will be even larger.

### Fractional atomic coordinates and isotropic or equivalent isotropic displacement parameters (Å^2^)

**Table:**

	*x*	*y*	*z*	*U*_iso_*/*U*_eq_	
Cu1	0.70192 (3)	0.10898 (3)	0.092940 (14)	0.03511 (11)	
S1	0.56142 (6)	−0.14977 (7)	−0.11161 (3)	0.04101 (16)	
S2	0.72370 (6)	0.27895 (8)	0.33102 (3)	0.04644 (17)	
O1	0.96149 (19)	−0.1681 (2)	0.29467 (12)	0.0631 (6)	
O2	0.96200 (17)	0.76310 (19)	0.03689 (11)	0.0528 (5)	
O3	0.91522 (15)	0.06510 (18)	0.06824 (9)	0.0398 (4)	
H3A	0.9289	−0.0271	0.0537	0.048*	
H3B	0.9461	0.1164	0.0342	0.048*	
N1	0.70065 (17)	−0.0949 (2)	0.14278 (10)	0.0354 (4)	
N2	0.8973 (2)	−0.3974 (3)	0.32101 (14)	0.0633 (7)	
H2A	0.9542	−0.4107	0.3519	0.076*	
H2B	0.8453	−0.4670	0.3133	0.076*	
N3	0.72932 (17)	0.3169 (2)	0.04894 (10)	0.0349 (4)	
N4	1.0166 (2)	0.5956 (2)	0.12020 (13)	0.0503 (6)	
H4A	1.0755	0.6508	0.1346	0.060*	
H4B	1.0036	0.5107	0.1402	0.060*	
N5	0.65964 (18)	0.0179 (2)	0.00029 (10)	0.0391 (4)	
N6	0.7224 (2)	0.2002 (2)	0.18720 (11)	0.0467 (5)	
C1	0.7870 (2)	−0.1250 (3)	0.19112 (12)	0.0351 (5)	
H1A	0.8483	−0.0554	0.1981	0.042*	
C2	0.7894 (2)	−0.2552 (3)	0.23123 (12)	0.0345 (5)	
C3	0.6977 (2)	−0.3586 (3)	0.22028 (13)	0.0394 (5)	
H3C	0.6965	−0.4475	0.2460	0.047*	
C4	0.6086 (2)	−0.3276 (3)	0.17083 (15)	0.0451 (6)	
H4C	0.5462	−0.3952	0.1629	0.054*	
C5	0.6128 (2)	−0.1952 (3)	0.13314 (13)	0.0394 (5)	
H5A	0.5523	−0.1751	0.0998	0.047*	
C6	0.8898 (2)	−0.2704 (3)	0.28559 (13)	0.0404 (5)	
C7	0.6561 (2)	0.3698 (3)	−0.00225 (14)	0.0429 (6)	
H7A	0.5926	0.3104	−0.0182	0.052*	
C8	0.6714 (3)	0.5085 (3)	−0.03205 (15)	0.0508 (7)	
H8A	0.6180	0.5431	−0.0668	0.061*	
C9	0.7665 (2)	0.5957 (3)	−0.00991 (14)	0.0447 (6)	
H9A	0.7792	0.6889	−0.0305	0.054*	
C10	0.8437 (2)	0.5437 (2)	0.04359 (12)	0.0340 (5)	
C11	0.8209 (2)	0.4028 (2)	0.07201 (12)	0.0335 (5)	
H11A	0.8710	0.3669	0.1082	0.040*	
C12	0.9465 (2)	0.6412 (2)	0.06743 (14)	0.0379 (5)	
C13	0.6193 (2)	−0.0526 (2)	−0.04580 (12)	0.0326 (5)	
C14	0.7230 (2)	0.2317 (2)	0.24690 (13)	0.0353 (5)	

### Atomic displacement parameters (Å^2^)

**Table:**

	*U*^11^	*U*^22^	*U*^33^	*U*^12^	*U*^13^	*U*^23^
Cu1	0.04718 (19)	0.02743 (16)	0.03065 (17)	−0.00594 (12)	−0.00671 (12)	0.00229 (10)
S1	0.0488 (4)	0.0379 (3)	0.0363 (3)	−0.0021 (3)	−0.0041 (3)	−0.0064 (2)
S2	0.0540 (4)	0.0510 (4)	0.0343 (3)	0.0064 (3)	−0.0001 (3)	−0.0033 (3)
O1	0.0675 (13)	0.0435 (11)	0.0777 (15)	−0.0095 (10)	−0.0353 (11)	0.0047 (10)
O2	0.0603 (12)	0.0309 (9)	0.0673 (13)	−0.0082 (8)	0.0081 (10)	0.0094 (8)
O3	0.0437 (9)	0.0324 (8)	0.0432 (9)	−0.0031 (7)	0.0032 (7)	0.0007 (7)
N1	0.0378 (10)	0.0320 (10)	0.0364 (10)	−0.0048 (8)	−0.0049 (8)	0.0045 (8)
N2	0.0633 (16)	0.0509 (15)	0.0754 (18)	−0.0085 (12)	−0.0333 (14)	0.0199 (12)
N3	0.0431 (11)	0.0284 (9)	0.0331 (10)	−0.0021 (8)	−0.0024 (8)	0.0022 (8)
N4	0.0463 (12)	0.0393 (12)	0.0653 (15)	−0.0105 (10)	−0.0087 (11)	0.0048 (10)
N5	0.0452 (11)	0.0387 (11)	0.0333 (10)	−0.0053 (9)	−0.0055 (8)	−0.0001 (8)
N6	0.0654 (14)	0.0382 (11)	0.0364 (12)	−0.0099 (10)	−0.0068 (10)	0.0004 (9)
C1	0.0358 (12)	0.0320 (11)	0.0376 (12)	−0.0039 (9)	−0.0038 (9)	0.0025 (9)
C2	0.0369 (12)	0.0320 (11)	0.0346 (12)	0.0016 (9)	0.0000 (9)	−0.0008 (9)
C3	0.0448 (13)	0.0309 (12)	0.0423 (13)	−0.0025 (10)	−0.0019 (10)	0.0066 (10)
C4	0.0436 (13)	0.0375 (13)	0.0541 (15)	−0.0115 (11)	−0.0082 (11)	0.0054 (11)
C5	0.0392 (12)	0.0364 (12)	0.0425 (13)	−0.0037 (10)	−0.0089 (10)	0.0043 (10)
C6	0.0431 (13)	0.0376 (13)	0.0405 (13)	0.0050 (10)	−0.0055 (10)	−0.0007 (10)
C7	0.0502 (14)	0.0362 (13)	0.0423 (14)	−0.0020 (11)	−0.0104 (11)	0.0031 (10)
C8	0.0602 (16)	0.0420 (14)	0.0498 (15)	0.0032 (12)	−0.0162 (13)	0.0090 (12)
C9	0.0555 (15)	0.0311 (12)	0.0475 (15)	0.0021 (11)	−0.0011 (12)	0.0092 (10)
C10	0.0392 (12)	0.0259 (10)	0.0368 (12)	0.0032 (9)	0.0061 (9)	−0.0006 (9)
C11	0.0379 (12)	0.0264 (11)	0.0362 (12)	−0.0002 (9)	−0.0012 (9)	0.0028 (9)
C12	0.0384 (12)	0.0263 (11)	0.0492 (14)	−0.0007 (9)	0.0114 (10)	−0.0009 (10)
C13	0.0363 (11)	0.0287 (11)	0.0329 (11)	0.0008 (9)	0.0018 (9)	0.0049 (9)
C14	0.0402 (12)	0.0270 (11)	0.0386 (13)	−0.0017 (9)	−0.0039 (10)	0.0040 (9)

### Geometric parameters (Å, °)

**Table:**

Cu1—N6	1.955 (2)	N5—C13	1.156 (3)
Cu1—N5	1.969 (2)	N6—C14	1.151 (3)
Cu1—N1	2.049 (2)	C1—C2	1.386 (3)
Cu1—N3	2.058 (2)	C1—H1A	0.9300
Cu1—O3	2.442 (4)	C2—C3	1.389 (3)
S1—C13	1.635 (2)	C2—C6	1.509 (3)
S2—C14	1.629 (3)	C3—C4	1.377 (3)
O1—C6	1.223 (3)	C3—H3C	0.9300
O2—C12	1.244 (3)	C4—C5	1.380 (3)
O3—H3A	0.8821	C4—H4C	0.9300
O3—H3B	0.8574	C5—H5A	0.9300
N1—C5	1.336 (3)	C7—C8	1.372 (4)
N1—C1	1.340 (3)	C7—H7A	0.9300
N2—C6	1.318 (3)	C8—C9	1.373 (4)
N2—H2A	0.8600	C8—H8A	0.9300
N2—H2B	0.8600	C9—C10	1.393 (3)
N3—C7	1.337 (3)	C9—H9A	0.9300
N3—C11	1.342 (3)	C10—C11	1.392 (3)
N4—C12	1.317 (3)	C10—C12	1.501 (3)
N4—H4A	0.8600	C11—H11A	0.9300
N4—H4B	0.8600		
			
N6—Cu1—N5	172.90 (9)	C4—C3—H3C	120.5
N6—Cu1—N1	87.87 (9)	C2—C3—H3C	120.5
N5—Cu1—N1	91.70 (9)	C3—C4—C5	119.3 (2)
N6—Cu1—N3	88.06 (9)	C3—C4—H4C	120.3
N5—Cu1—N3	93.28 (8)	C5—C4—H4C	120.3
N1—Cu1—N3	171.32 (8)	N1—C5—C4	122.4 (2)
O3—Cu1—N1	87.25 (7)	N1—C5—H5A	118.8
O3—Cu1—N3	85.68 (7)	C4—C5—H5A	118.8
O3—Cu1—N5	89.57 (7)	O1—C6—N2	122.5 (2)
O3—Cu1—N6	97.49 (8)	O1—C6—C2	120.1 (2)
H3A—O3—H3B	101.7	N2—C6—C2	117.4 (2)
C5—N1—C1	118.2 (2)	N3—C7—C8	122.4 (2)
C5—N1—Cu1	122.97 (16)	N3—C7—H7A	118.8
C1—N1—Cu1	118.66 (15)	C8—C7—H7A	118.8
C6—N2—H2A	120.0	C7—C8—C9	119.2 (2)
C6—N2—H2B	120.0	C7—C8—H8A	120.4
H2A—N2—H2B	120.0	C9—C8—H8A	120.4
C7—N3—C11	118.8 (2)	C8—C9—C10	119.6 (2)
C7—N3—Cu1	121.07 (16)	C8—C9—H9A	120.2
C11—N3—Cu1	120.14 (15)	C10—C9—H9A	120.2
C12—N4—H4A	120.0	C11—C10—C9	117.7 (2)
C12—N4—H4B	120.0	C11—C10—C12	123.5 (2)
H4A—N4—H4B	120.0	C9—C10—C12	118.8 (2)
C13—N5—Cu1	166.17 (19)	N3—C11—C10	122.3 (2)
C14—N6—Cu1	167.6 (2)	N3—C11—H11A	118.9
N1—C1—C2	123.1 (2)	C10—C11—H11A	118.9
N1—C1—H1A	118.5	O2—C12—N4	122.2 (2)
C2—C1—H1A	118.5	O2—C12—C10	118.7 (2)
C1—C2—C3	118.0 (2)	N4—C12—C10	119.1 (2)
C1—C2—C6	116.9 (2)	N5—C13—S1	179.0 (2)
C3—C2—C6	125.1 (2)	N6—C14—S2	179.1 (2)
C4—C3—C2	119.1 (2)		

### Hydrogen-bond geometry (Å, °)

**Table:**

*D*—H···*A*	*D*—H	H···*A*	*D*···*A*	*D*—H···*A*
O3—H3A···O2^i^	0.88	1.94	2.815 (3)	171
O3—H3B···O2^ii^	0.86	2.00	2.848 (3)	172
N2—H2A···O3^iii^	0.86	2.09	2.944 (3)	176
N4—H4B···O1^iv^	0.86	2.05	2.857 (3)	157

Symmetry codes: (i) *x*, *y*−1, *z*; (ii) −*x*+2, −*y*+1, −*z*; (iii) −*x*+2, *y*−1/2, −*z*+1/2; (iv) −*x*+2, *y*+1/2, −*z*+1/2.
